# The host antiviral protein SAMHD1 suppresses NF-κB activation by interacting with the IKK complex during inflammatory responses and viral infection

**DOI:** 10.1016/j.jbc.2023.104750

**Published:** 2023-04-24

**Authors:** Hua Yang, Constanza E. Espada, Stacia Phillips, Nicholas Martinez, Adam D. Kenney, Jacob S. Yount, Yong Xiong, Li Wu

**Affiliations:** 1 Department of Microbiology and Immunology, Carver College of Medicine, The University of Iowa, Iowa City, Iowa, USA; 2 Department of Molecular Biophysics and Biochemistry, Yale University, New Haven, Connecticut, USA; 3 Department of Microbial Infection and Immunity, The Ohio State University, Columbus, Ohio, USA

**Keywords:** SAMHD1, NF-κB, IKKα, IKKβ, IKKγ, TAK1, phosphorylation, immunoprecipitation, THP-1 cells, viral infection

## Abstract

Sterile alpha motif and histidine-aspartate (HD) domain–containing protein 1 (SAMHD1) inhibits HIV-1 replication in nondividing cells by reducing the intracellular dNTP pool. SAMHD1 also suppresses NF-κB activation induced by inflammatory stimuli and viral infections. Specifically, SAMHD1-mediated reduction of NF-κB inhibitory protein (IκBα) phosphorylation is important for the suppression of NF-κB activation. However, while the inhibitors of NF-κB kinase subunit alpha and beta (IKKα and IKKβ) regulate IκBα phosphorylation, the mechanism by which SAMHD1 regulates phosphorylation of IκBα remains unclear. Here, we report that SAMHD1 suppresses phosphorylation of IKKα/β/γ *via* interaction with IKKα and IKKβ, thus inhibiting subsequent phosphorylation of IκBα in monocytic THP-1 cells and differentiated nondividing THP-1 cells. We show that knockout of SAMHD1 enhanced phosphorylation of IKKα, IKKβ, and IKKγ in THP-1 cells treated with the NF-κB activator lipopolysaccharide or infected with Sendai virus and SAMHD1 reconstitution inhibited phosphorylation of IKKα/β/γ in Sendai virus–infected THP-1 cells. We demonstrate that endogenous SAMHD1 interacted with IKKα and IKKβ in THP-1 cells and recombinant SAMHD1 bound to purified IKKα or IKKβ directly *in vitro*. Mapping of these protein interactions showed that the HD domain of SAMHD1 interacts with both IKKα and IKKβ and that the kinase domain of IKKα and the ubiquitin-like domain of IKKβ are required for their interactions with SAMHD1, respectively. Moreover, we found that SAMHD1 disrupts the interaction between upstream kinase TAK1 and IKKα or IKKβ. Our findings identify a new regulatory mechanism by which SAMHD1 inhibits phosphorylation of IκBα and NF-κB activation.

SAMHD1 is a deoxynucleoside triphosphate triphosphohydrolase (dNTPase) that reduces the concentration of intracellular dNTPs (1, 2). SAMHD1 is a cellular restriction factor of human immunodeficiency virus type 1 (HIV-1) replication in nondividing myeloid cells (3, 4) and resting CD4+ T cells (5, 6). SAMHD1 also inhibits the replication of other viral pathogens, including herpesviruses (7, 8), enterovirus 71, hepatitis B virus, and hepatitis C virus (9, 10, 11). The Aicardi–Goutières syndrome, an autoinflammatory disorder, influences the brain, skin, and immune system and is associated with mutations in SAMHD1 that regulate cellular dNTP and RNA homeostasis (12, 13). We have reported that SAMHD1 suppresses NF-κB activation and type I interferon (IFN-I) activation induced by inflammatory stimuli and viral infections, suggesting that SAMHD1 plays a significant role in modulating innate immunity (14, 15). SAMHD1 also regulates stalled DNA replication forks and reduces the accumulation of cytosolic single-stranded DNA, which leads to a decrease in the production of IFN-I through the cGAS-STING pathway (16). These studies suggest multifaceted functions of SAMHD1 in regulating innate immunity.

NF-κB is activated through two distinct signaling pathways defined as canonical and noncanonical (17). In the canonical pathway and in the absence of stimulation, IκBα is bound to NF-κB family proteins p65 and p50 to prevent the p65/p50 heterodimer from translocating to the nucleus and activating gene transcription. Upon inflammatory stimulation or viral infection, the IκBα residues Ser32 and Ser36 are phosphorylated by a homo- or heterodimer of IKKα and IKKβ (17). Phosphorylation of these residues leads to IκBα ubiquitination and subsequent proteasomal degradation. The released p65/p50 heterodimer is then free to translocate to the nucleus to activate NF-κB target gene transcription (18). Thus, the phosphorylation of IκBα is critical for NF-κB activation.

The IKK complex consists of two catalytic subunits IKKα/IKKβ and regulatory subunit IKKγ (also called NF-κB essential modulator, or NEMO). Both IKKα and IKKβ contain an N-terminal kinase domain (KD), a ubiquitin-like domain (ULD), a scaffold dimerization domain (SDD), and a C-terminal NEMO-binding domain (19, 20, 21). The phosphorylation of serine residues in the KD, which is induced by transforming growth factor β–activated kinase 1 (TAK1), is essential to activate the catalytic activity of IKKα and IKKβ (22). TAK1 phosphorylates IKKβ at Ser177, which permits IKKβ to autophosphorylate itself at Ser181 (23). Although IKKγ does not have kinase activity, it is essential for modulating activation of IKKα and IKKβ (24). IKKγ is phosphorylated at Ser376 by IKKβ, and phosphorylation of IKKγ plays an important regulatory role in activation of IKKβ and NF-κB (25). In the noncanonical NF-κB pathway, the phosphorylation of IKKα is induced by NF-κB-inducing kinase, leading to phosphorylation and degradation of p100 and nuclear translocation of p52 (26, 27).

Our previous study showed that SAMHD1 interacts with NF-κB1/p50 and NF-κB2/p52 and reduces phosphorylation of IκBα, thereby inhibiting NF-κB activation (14). We also reported that SAMHD1 inhibits NF-κB activation mediated by TAK-1 and tumor necrosis factor (TNF) receptor associated factor 6 (TRAF6), two key proteins of the NF-κB signaling pathway that are upstream of IκBα (28). However, the exact mechanism of SAMHD1 regulation of IκBα phosphorylation remains unclear.

In this study, we found that SAMHD1 inhibited IKKα- and IKKβ-mediated NF-κB activation in cells upon viral infection or inflammatory stimulation. SAMHD1 negatively affected phosphorylation of IKKα, IKKβ, and IKKγ in THP-1 cells treated with lipopolysaccharide (LPS) or infected with Sendai virus (SeV). We demonstrated that SAMHD1 interacted with IKKα and IKKβ in THP-1 cells and HEK293T cells. Furthermore, we identified that the HD domain of SAMHD1, the KD of IKKα, and the ULD of IKKβ were involved in the interaction between SAMHD1 and IKKα or IKKβ. Furthermore, we observed that SAMHD1 overexpression disrupted the interaction between TAK1 and IKKα or IKKβ. Overall, our findings reveal a new regulatory mechanism by which SAMHD1 inhibits NF-κB activation during viral infection and inflammation.

## Results

### SAMHD1 suppresses IKKα- and IKKβ-mediated NF-κB activation in HEK293T cells

**Figure 1:**
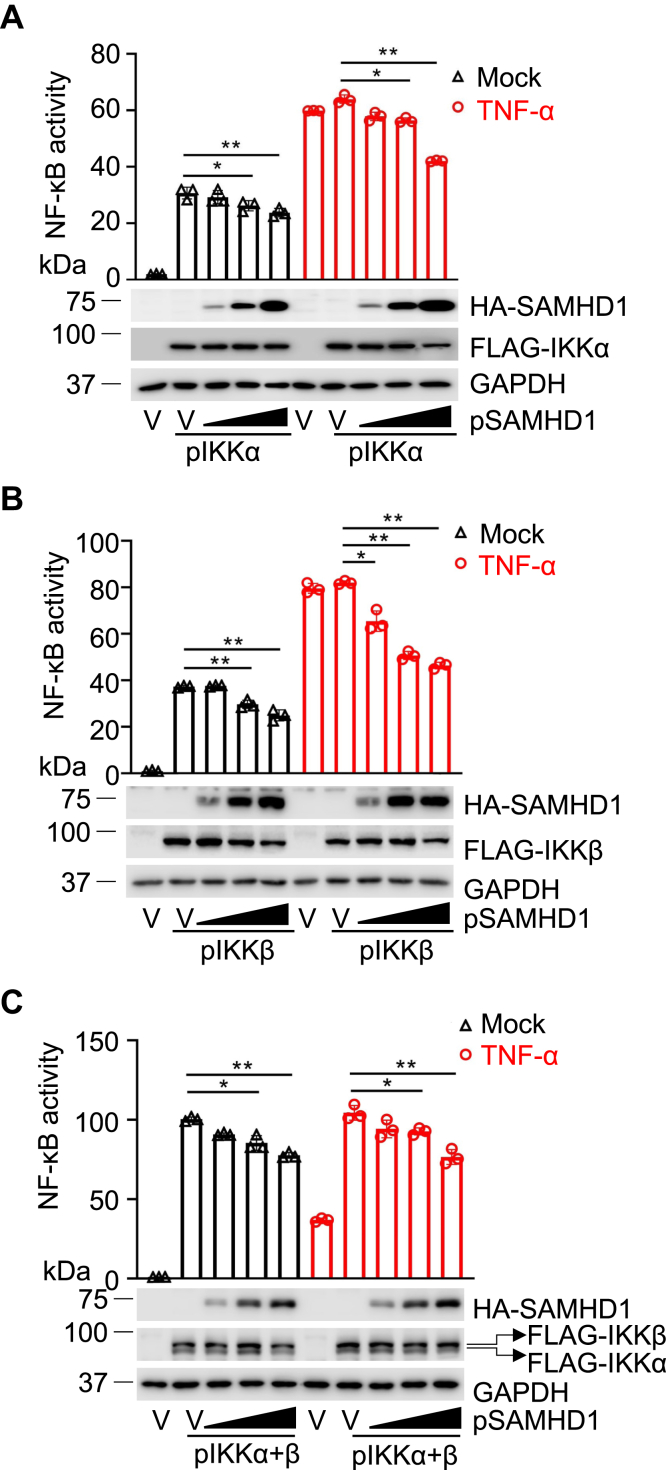
**SAMHD1 suppresses IKKα- and IKKβ-mediated NF-κB activation in the absence or presence of TNF-α.** HEK293T cells were cotransfected with 50 ng pN3-3 × FLAG-IKKα (*A*), or 25 ng pN3-3 × FLAG-IKKβ (*B*), or 50 ng pN3-3 × FLAG-IKKα and 25 ng pN3-3 × FLAG-IKKβ (*C*), the increased amounts of pRK-HA-SAMHD1, pNF-κB-luciferase (50 ng), and TK-renilla (10 ng). An empty vector was used to maintain the same amount of plasmid DNA in each transfection. At 24 h post transfection, cells were treated with TNF-α (10 ng/ml) for 2 h and then luciferase assays were performed. Results are expressed relative to empty vector, untreated cells, which are set to 1. The *t* test was used for statistical significance. ∗*p* < 0.05; ∗∗*p* < 0.01 (compared with vector control, lane 2 or 7 in each group). The expression levels of indicated proteins were detected by Western blot and GAPDH was a loading control. V, empty vector control.

We previously reported that SAMHD1 suppresses NF-κB activation by interacting with p50/p52 and by reducing phosphorylation of IκBα (14). However, the underlying molecular mechanisms remain to be defined. To explore how SAMHD1 inhibits NF-κB activation, we first examined whether SAMHD1 could inhibit IKKα- and IKKβ-mediated activation of an NF-κB reporter gene. HEK293T cells were cotransfected with plasmids expressing an NF-κB luciferase reporter with increasing amounts of SAMHD1 and IKKα (Fig. 1*A*), IKKβ (Fig. 1*B*), or both IKKα and IKKβ (Fig. 1*C*). When HEK293T cells overexpressed IKKα, IKKβ, or both, a significant increase in luciferase expression from the NF-κB reporter was observed (Fig. 1, *A*–*C*, compare lane 1 with 2 in the mock-treated groups). Treatment of cells with the inflammatory cytokine TNF-α also significantly induces NF-κB activation (14). Indeed, TNF-α treatment resulted in stronger activation of reporter expression to levels that were not further augmented by expression of IKKα or IKKβ alone (Fig. 1, *A* and *B*, lane 6 and 7). SAMHD1 inhibited IKKα- or IKKβ-mediated NF-κB activation in a dose-dependent manner in the absence or presence of TNF-α (Fig. 1, *A* and *B*). When IKKα and IKKβ were coexpressed in HEK293T cells, we observed synergistic enhancement of the NF-κB activity as expected, which was not further promoted by TNF-α treatment (Fig. 1*C*, compare lanes 2 and 7), likely due to saturated NF-κB activation. Interestingly, SAMHD1 inhibited both IKKα- and IKKβ-mediated NF-κB activation in a dose-dependent manner regardless of TNF-α treatment (Fig. 1*C*). These results indicate that exogenous SAMHD1 suppresses IKKα- and IKKβ-mediated NF-κB activation in HEK293T cells.

### SAMHD1 inhibits phosphorylation of IKKα/β/γ induced by LPS treatment

**Figure 2:**
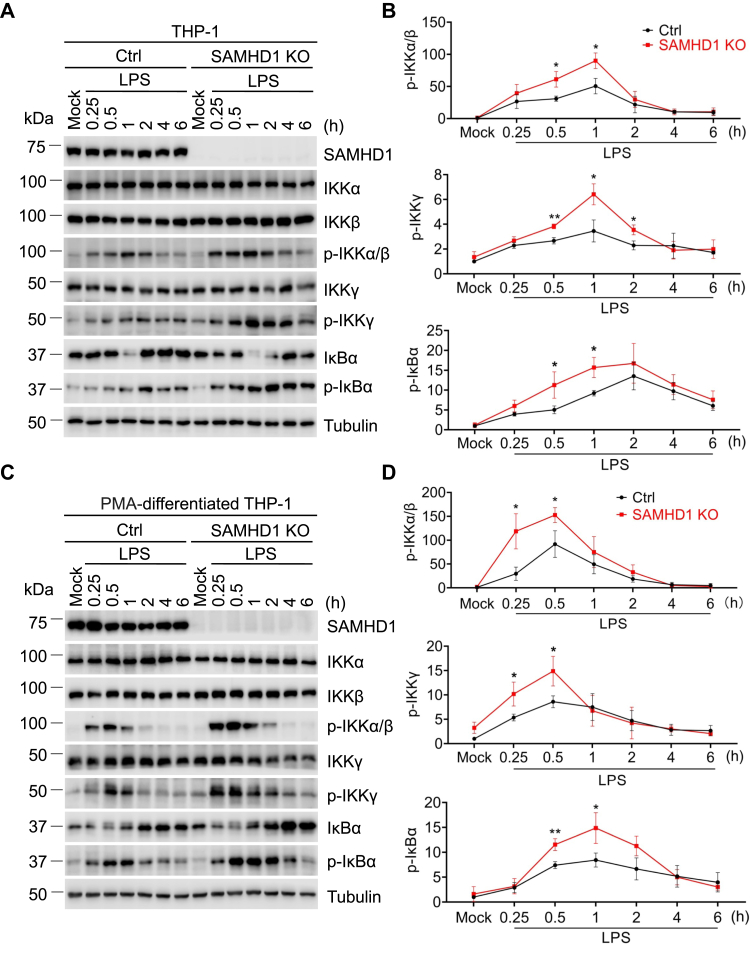
**SAMHD1 inhibits phosphorylation of IKKα/β/γ induced by LPS treatment.***A* and *C*, THP-1 control (Ctrl) cells and THP-1 SAMHD1 knockout (KO) cells (*A*) or PMA-differentiated THP-1 ctrl cells and PMA-differentiated THP-1 SAMHD1 KO cells (*C*) were treated with LPS (100 ng/ml) for 15 min to 6 h or mock treated. The cell lysates were harvested at each time point and endogenous SAMHD1, IKKα/β/γ, p- IKKα/β, p-IKKγ, IκBα, p- IκBα, and tubulin were detected by Western blot. Tubulin was a loading control. *B* and *D*, The relative p-IKKα/β, p-IKKγ, and p-IκBα levels were quantified by densitometry analysis. Relative p-IKKα/β, p-IKKγ, and p-IκBα levels were normalized to tubulin to avoid the difference of total protein expression levels due to different treatments. The results were presented as means ± SD. Levels of each phosphoprotein are expressed relative to THP-1 control cells without LPS treatment, which were set to 1. The *t* test was used for statistical significance compared with THP-1 control cells. ∗*p* < 0.05; ∗∗*p* < 0.01. The data shown in *B* and *D* represent three independent experiments.

We previously reported that LPS-induced phosphorylation of IκBα is increased in THP-1 SAMHD1 knockout (KO) cells compared with THP-1 control cells (14). To investigate the mechanisms by which SAMHD1 inhibits phosphorylation of IκBα, the expression and phosphorylation levels of IKKα/β/γ were measured in THP-1 cells over a time course of LPS treatment. The total levels of IKKα, IKKβ, and IKKγ expression were not significantly affected by LPS treatment. Phosphorylation of IKKα/β and IKKγ significantly increased between 15 min and 1 h after LPS treatment (Fig. 2, *A* and *B*). Because the phosphor-specific antibody detected both IKKα and IKKβ phosphorylation (p-IKKα/β), we labeled and quantified p-IKKα/β together (Fig. 2, *A*–*D*). After 1 h of LPS treatment, the phosphorylation of IKKα/β and IKKγ gradually decreases to baseline levels. We found that the level of p-IKKα/β and phosphorylation of IKKγ (p-IKKγ) was higher in THP-1 SAMHD1 KO cells compared with the THP-1 control cells (Fig. 2, *A*–*D*). Other NF-κB activators, TNF-α and interleukin 1 beta (IL-1β), were also tested and showed similar results (Fig. S1). Together, these data demonstrate that endogenous SAMHD1 suppresses the phosphorylation of IKKα/β and IKKγ in THP-1 cells in response to inflammatory stimuli.

Our previous studies suggested that the function of SAMHD1 is dependent on cell differentiation (14, 29). For example, the dNTPase activity of SAMHD1 is not necessary for its inhibition of NF-κB and IFN-I activation in dividing HEK293T cells, while the suppression function of SAMHD1 is dependent on its dNTPase in phorbol 12-myristate 13-acetate (PMA)-differentiated nondividing monocytic cells (14, 29). Thus, we utilized PMA to induce THP-1 cells into nondividing macrophage-like cells and performed similar experiments. To test whether PMA could affect phosphorylation of IKKα/β during cell differentiation, THP-1 control cells and THP-1 SAMHD1 KO cells were treated with PMA (30 ng/ml) for 1 to 48 h or mock treated and then subjected to immunoblot analysis. PMA slightly induces phosphorylation of IKKα/β between 1 h and 8 h in THP-1 control cells and THP-1 SAMHD1 KO cells. After 24 h and 48 h of PMA treatment, the p-IKKα/β was hardly detectable in the absence of inflammatory stimuli (Fig. S2).

To confirm the effect of SAMHD1 in NF-κB activation in nondividing cells, the phosphorylation levels of IKKα/β/γ were measured in PMA-differentiated THP-1 cells (Fig. 2*C*). The total expression levels of IKKα, IKKβ, and IKKγ were not significantly influenced by LPS treatment in the differentiated THP-1 cells. The phosphorylation of IKKα/β and IKKγ was induced by LPS 15 min to 30 min post treatment in PMA-differentiated THP-1 cells, after which levels of p-IKKα/β and p-IKKγ returned to baseline (Fig. 2*C*). The peak of p-IKKα/β and p-IKKγ in PMA-differentiated cells appeared earlier than that observed in dividing THP-1 cells, suggesting that noncycling THP-1 cells were more sensitive to LPS treatment. The levels of p-IKKα/β and p-IKKγ in the THP-1 control cells were lower than that in THP-1 SAMHD1 KO cells (Fig. 2, *C* and *D*). These results further corroborate that endogenous SAMHD1 inhibits phosphorylation of IKKα/β/γ induced by LPS treatment.

### SAMHD1 inhibits phosphorylation of IKKα/β/γ induced by SeV infection

**Figure 3:**
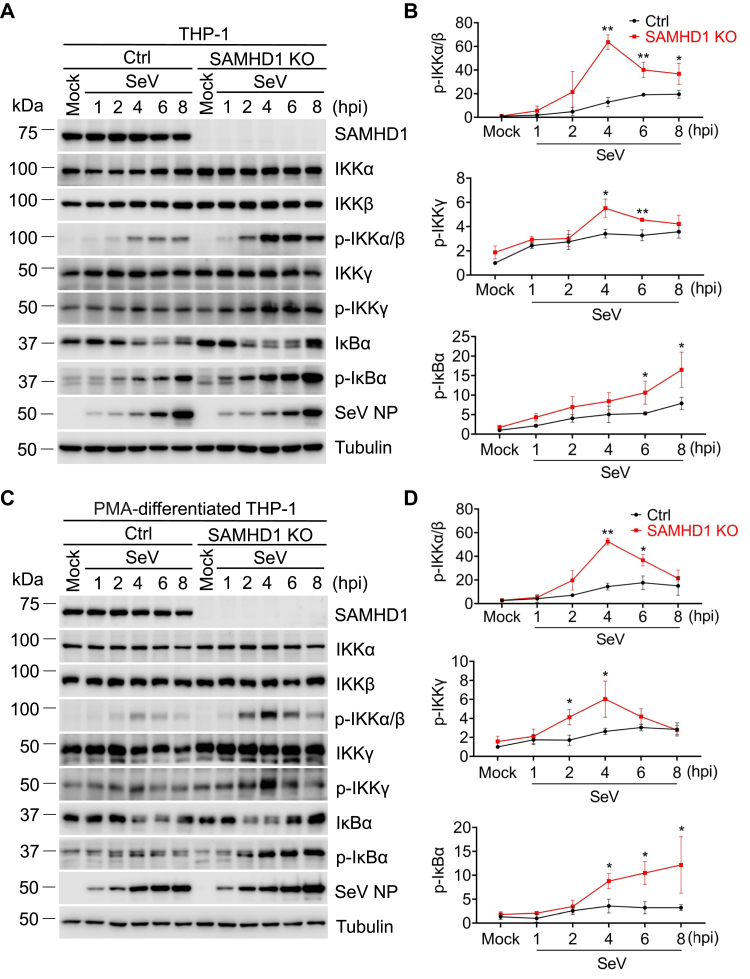
**SAMHD1 inhibits phosphorylation of IKKα/β/γ induced by SeV infection.***A* and *C*, THP-1 control cells and THP-1 SAMHD1 KO cells (*A*) or PMA-differentiated THP-1 control cells and PMA-differentiated SAMHD1 KO cells (*C*) were infected with SeV (multiplicity of infection [MOI] of 10) for 1 to 8 h or mock treated. The expression levels of SAMHD1, IKKα/β/γ, p- IKKα/β, p-IKKγ, IκBα, p- IκBα, SeV nucleoprotein (NP) protein, and tubulin were measured by Western blot. Tubulin was used as a loading control. *B* and *D*, The relative p-KKα/β, p-IKKγ, and p-IκBα levels were quantified by densitometry analysis. Relative p-KKα/β, p-IKKγ, and p-IκBα levels were normalized to tubulin. Levels of each phosphoprotein are expressed relative to THP-1 control cells without SeV infection which were set to 1. The *t* test was used for statistical significance compared with the THP-1 control cells. ∗*p* < 0.05; ∗∗*p* < 0.01. The data shown in *B* and *D* represent three independent experiments.

We found that endogenous SAMHD1 inhibits phosphorylation of IKKα/β/γ induced by inflammatory stimuli (Fig. 2). To examine the role of SAMHD1 in inhibiting NF-κB in response to virus infection in dividing and nondividing cells, we infected THP-1 cells with SeV, which is known to efficiently activate NF-κB signaling (14, 29). As expected, the expression level of SeV nucleoprotein increased over time, demonstrating successful viral replication (Fig. 3, *A* and *C*). Although the total levels of IKKα, IKKβ, and IKKγ were not significantly influenced by SeV infection, the level of p-IKKα/β and p-IKKγ increased in response to infection and the peak of p-IKKα/β and p-IKKγ appeared at 4 h post infection (hpi) in THP-1 cells (Fig. 3, *A* and *B*). At 4 hpi, the p-IKKα/β and p-IKKγ in the SAMHD1 KO cells were nearly 4- and 2-fold higher relative to THP-1 control cells (Fig. 3, *A* and *B*), demonstrating that SAMHD1 suppresses phosphorylation of IKKα/β and IKKγ induced by SeV infection. Similar results were overserved in PMA-differentiated THP-1 cells (Fig. 3, *C* and *D*), indicating that endogenous SAMHD1 inhibits virus-induced phosphorylation of IKKα/β/γ in both cycling and noncycling cells.

### SAMHD1 reconstitution inhibits phosphorylation of IKKα/β/γ induced by SeV infection

**Figure 4:**
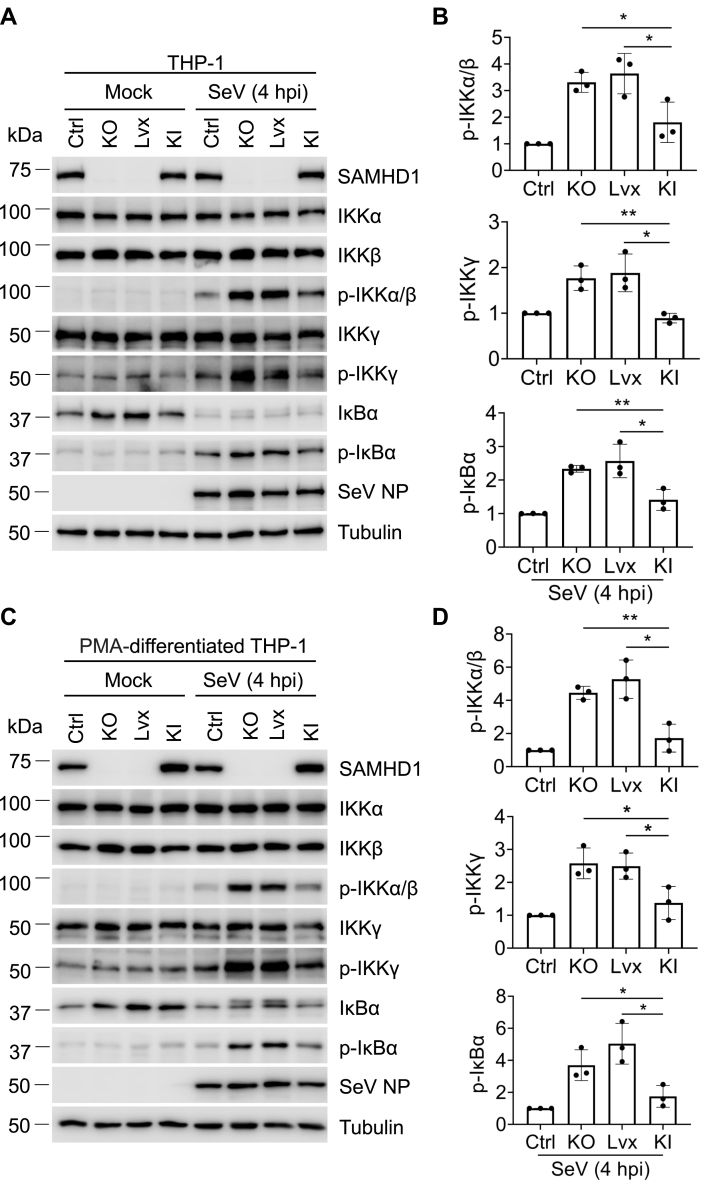
**SAMHD1 reconstitution inhibits phosphorylation of IKKα/β/γ induced by SeV infection.***A* and *C*, (*A*) THP-1 control cells, THP-1 SAMHD1 KO cells, THP-1 Lvx cells, and THP-1 SAMHD1 KI cells were infected with SeV (MOI = 10) for 4 h or mock treated. *C*, THP-1 control cells, THP-1 SAMHD1 KO cells, THP-1 Lvx cells, and THP-1 SAMHD1 KI cells were differentiated by PMA (30 ng/ml) for 48 h and then infected with SeV (MOI = 10) for 4 h or mock treated. The expression levels of SAMHD1, IKKα/β/γ, p- IKKα/β, p-IKKγ, IκBα, p-IκBα, SeV nucleoprotein (NP) protein, and tubulin were measured by Western blot. Tubulin was used as a loading control. *B* and *D*, The relative p-IKKα/β, p-IKKγ, and p-IκBα levels were quantified by densitometry analysis. The results were presented as means ± SD. Levels of each phosphoprotein are expressed relative to control cells infected with SeV for 4 h, which were set to 1. The *t* test was used for statistical significance. ∗*p* < 0.05; ∗∗*p* < 0.01. The data shown in *B* and *D* represent three independent experiments.

SAMHD1 knock-in (KI) cells were generated from parental THP-1 SAMHD1 KO cells to reconstitute the expression of SAMHD1 with THP-1 Lvx lentivirus (Lvx) cells serving as a control cell population (30). THP-1 control cells, THP-1 SAMHD1 KO cells, THP-1 Lvx cells, and THP-1 SAMHD1 KI cells were infected with SeV for 4 h or mock treated (Fig. 4). As expected, SAMHD1 protein was detected in the THP-1 control cells and THP-1 SAMHD1 KI cells but not in THP-1 SAMHD1 KO and THP-1 Lvx cells. The expression of total IKKα, IKKβ, and IKKγ in all cell lines was similar. At 4 h post SeV infection, the levels of phosphorylated IKKα/β and IKKγ in THP-1 SAMHD1 KO cells and THP-1 Lvx cells were higher than that observed in THP-1 control cells and THP-1 SAMHD1 KI cells (Fig. 4, *A* and *B*), confirming that SAMHD1 inhibits phosphorylation of IKKα/β and IKKγ in THP-1 cells. To confirm the role of SAMHD1 in inhibiting phosphorylation of IKKα/β/γ in nondividing cells, the cells were differentiated by PMA prior to SeV infection. As shown in Figure 4, *C* and *D*, differentiated THP-1 SAMHD1 KO cells and differentiated THP-1 Lvx cells lacking SAMHD1 expression promoted p-IKKα/β and p-IKKγ compared with THP-1 control cells and SAMHD1 KI cells. Thus, SAMHD1 reconstitution in SAMHD1 KO cells inhibits phosphorylation of IKKα/β/γ induced by SeV infection. These results also complement our loss-of-function studies.

### SAMHD1 interacts with IKKα and IKKβ

**Figure 5:**
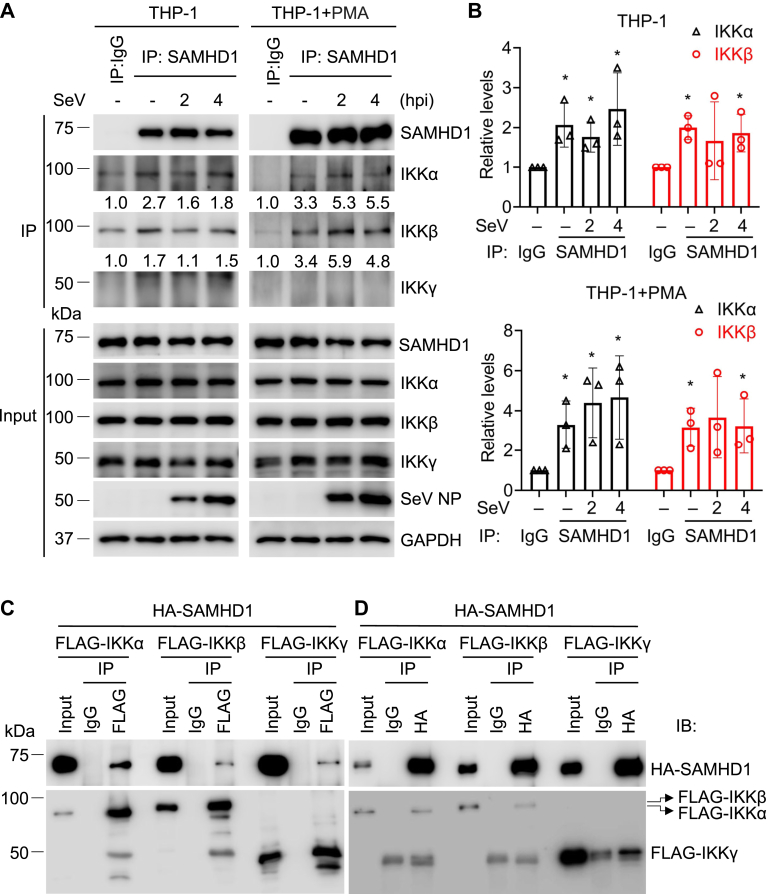
**SAMHD1 interacts with IKKα and IKKβ in cells.***A*, THP-1 control cells and PMA-differentiated THP-1 control cells were infected with SeV (MOI = 10) for indicated times or mock treated. Coimmunoprecipitation was performed with SAMHD1 antibody, Mouse IgG was used as a negative control. Input and immunoprecipitation (IP) samples were analyzed by Western blot. *B*, The relative levels of IKKα and IKKβ were quantified by densitometry analysis, and the IgG control was set as 1. The *t* test was used for statistical significance compared with IgG control. ∗*p* < 0.05. The data shown in *B* represent three independent experiments. *C* and *D*, HEK293T cells were cotransfected with plasmids encoding HA-SAMHD1 and indicated FLAG-IKKα (85 kDa), FLAG-IKKβ (87 kDa), or FLAG-IKKγ (48 kDa) separately. HEK293T cells were harvested after 48 h transfection. FLAG antibody (*C*) or HA antibody (*D*) was used for IP. The same amount of mouse IgG or rabbit IgG was used as a negative control. Input and IP samples were analyzed by immunoblotting (IB).

**Figure 6:**
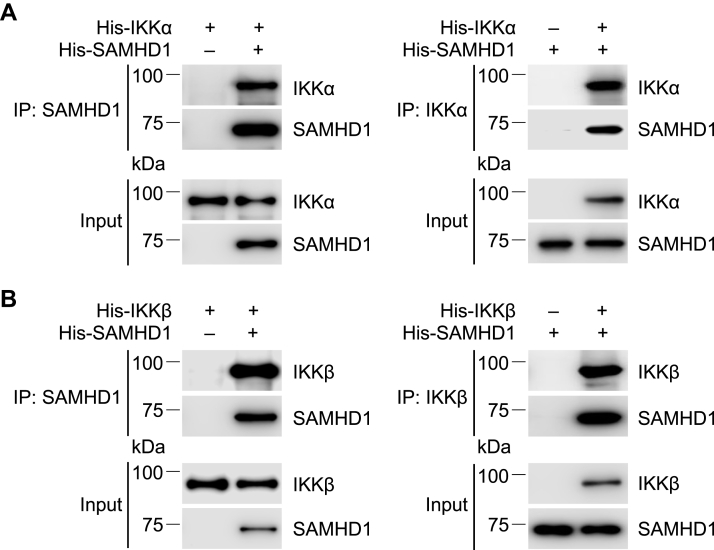
**Recombinant SAMHD1 interacts directly with purified rIKKα or IKKβ *in vitro*.***A*, His-SAMHD1 (5.6 μg) with or without His-IKKα (1 μg) was incubated with Dynabeads Protein A and SAMHD1 antibody (*left panel*). His-IKKα (5.6 μg) with or without His-SAMHD1 (1 μg) was incubated with Dynabeads Protein A and IKKα antibody (*right panel*). Input and IP samples were analyzed by Western blot. *B*, His-SAMHD1 (5.6 μg) with or without His-IKKβ (1 μg) was incubated with Dynabeads Protein A and SAMHD1 antibody (*left panel*). His-IKKβ (5.6 μg) with or without His-SAMHD1 (1 μg) was incubated with Dynabeads Protein A and IKKβ antibody (*right panel*). Input and immunoprecipitation (IP) samples were analyzed by Western blot.

We previously found that endogenous SAMHD1 interacts with key proteins like p50 and IκBα and inhibits NF-κB activation (14). To explore the mechanism of SAMHD1-mediated inhibition of p-IκBα, we tested whether endogenous SAMHD1 interacts with IKKα/β/γ in THP-1 cells that were mock treated or infected with SeV. Endogenous SAMHD1 interacted with IKKα and IKKβ but did not coimmunoprecipitate with IKKγ with or without SeV infection (Fig. 5, *A* and *B*). Similar results were obtained after stimulation of THP-1 cells with LPS (Fig. S3). Next, HEK293T cells were cotransfected with plasmids expressing hemagglutinin (HA)-tagged SAMHD1 and FLAG-tagged IKKα, IKKβ, or IKKγ. A FLAG-antibody (Fig. 5*C*) or HA antibody (Fig. 5*D*) was used for immunoprecipitation (IP) and IgG was used as a negative control. Coimmunoprecipitation (Co-IP) confirmed that SAMHD1 interacted with exogenous IKKα, IKKβ, and IKKγ in HEK293T cells (Fig. 5, *C* and *D*). To determine whether the interaction between SAMHD1 and IKKα or IKKβ is direct, we performed an *in vitro* pull-down assay. As shown in Figure 6, *A* and *B*, recombinant SAMHD1 interacted directly with purified IKKα and IKKβ, suggesting a potential mechanism of SAMHD1-mediated inhibition of p-IκBα through the SAMHD1-IKKs complex.

### The HD domain of SAMHD1 is required for its interaction with IKKα and IKKβ

**Figure 7:**
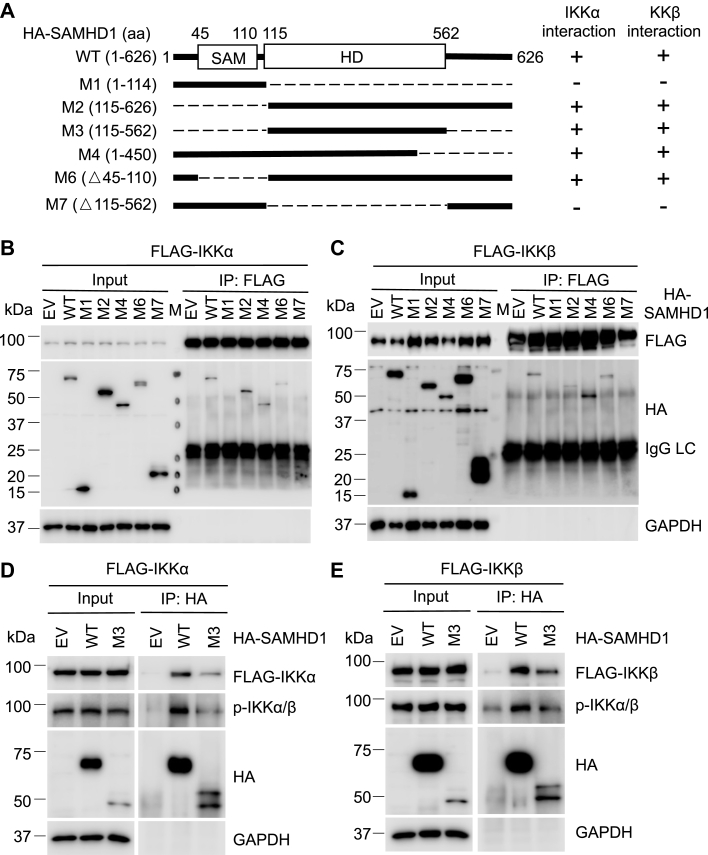
**The HD domain of SAMHD1 is required for its interaction with IKKα and IKKβ.***A*, schematic diagrams of SAMHD1 wildtype (WT) and truncated mutants (M1, M2, M3, M4, M6, and M7). *B* and *C*, HEK293T cells were cotransfected with plasmids expressing FLAG-IKKα (*B*), FLAG-IKKβ (*C*), or empty vector, HA-SAMHD1 WT or SAMHD1 truncated mutants (M1, M2, M4, M6, and M7) for 48 h. An empty vector was used to maintain the same amount of plasmid DNA in transfection. FLAG antibody was used for immunoprecipitation (IP). Input and IP samples were analyzed by Western blot. *D* and *E*, HEK293T cells were cotransfected with plasmids expressing FLAG-IKKα (*D*), FLAG-IKKβ (*E*), or empty vector, and HA-SAMHD1 WT or HA-SAMHD1 HD domain (M3) for 48 h. HA antibody was used for IP. Input and IP samples were analyzed by Western blot. FLAG antibody and p-IKKα/β antibody were used for immunoblotting. HD, histidine aspartic domain; IgG LC, IgG light chain; M, molecular weight marker; SAM, sterile alpha motif.

We reported that the HD domain of SAMHD1 interacts with IFN regulatory factor 7 (IRF7) and mediates suppression of IFN-I activation (14). To map the domains of SAMHD1 required for its interaction with IKKα and IKKβ, HEK293T cells were cotransfected with a series of plasmids expressing HA-SAMHD1 wildtype (WT) or truncated mutants (M1, M2, M4, M6, and M7) (14) and FLAG-IKKα or FLAG-IKKβ (Fig. 7*A*). Co-IP results showed that SAMHD1 M1 and M7 lacking the HD domain did not interact with IKKα or IKKβ, suggesting that the HD domain of SAMHD1 is required for its interaction with IKKα and IKKβ (Fig. 7, *B* and *C*). Because of lower expression of the HD domain alone (M3) compared with other mutants (14), HEK293T cells were cotransfected in a separate experiment with empty vector or plasmids expressing SAMHD1 WT or the HD domain alone (M3) and IKKα or IKKβ. We found that the HD domain of SAMHD1 interacted with FLAG-IKKα and p-IKKα (active) (Fig. 7*D*) and FLAG-IKKβ and p-IKKβ (active) (Fig. 7*E*). Thus, the HD domain of SAMHD1 is necessary and sufficient for its interaction with IKKα and IKKβ.

### SAMHD1 C-terminal truncation suppresses IKKα- or IKKβ-mediated NF-κB activation

**Figure 8:**
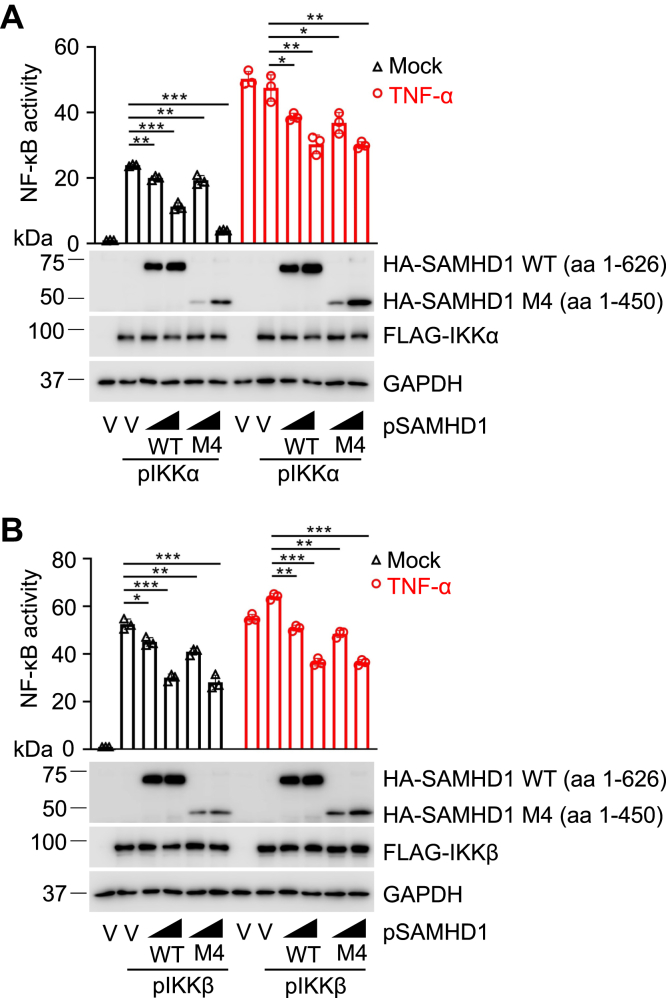
**SAMHD1 C-terminal truncation suppresses IKKα- or IKKβ-mediated NF-κB activation.** HEK293T cells were cotransfected with 50 ng of pN3-3 × FLAG-IKKα (*A*), or 25 ng of pN3-3 × FLAG-IKKβ (*B*), pRK-HA-SAMHD1 WT (200 ng or 400 ng, respectively) or pRK-HA-SAMHD1 M4 (200 ng or 400 ng, respectively), pNF-κB-luciferase (50 ng), and a plasmid expressing TK-renilla (10 ng). An empty vector was used to maintain the same amount of plasmid DNA in each transfection. At 24 h post transfection, cells were treated with TNF-α (10 ng/ml) for 2 h and then luciferase assays were performed. Results are expressed relative to empty vector, untreated cells, which are set to 1. The *t* test was used for statistical significance. ∗*p* < 0.05; ∗∗*p* < 0.01; ∗∗∗*p* < 0.001 (compared with vector control, lane 2 or 8 in each group). The expression levels of indicated proteins were detected by Western blot, and GAPDH was a loading control. V, empty vector control.

The above data indicate an important role of the HD domain of SAMHD1 in suppressing NF-κB activation (Fig. 7). Consistently, our published results demonstrated that SAMHD1-mediated suppression of NF-κB activation requires its dNTPase activity that depends on the HD domain of SAMHD1 (29). Our previous studies also identified that a cyclin-binding motif (aa 450–455) in the C terminus of SAMHD1 regulates protein phosphorylation, localization, and stability (31). However, the role of the C terminus of SAMHD1 in regulating NF-κB activation is unknown. To address this question, we selected SAMHD1 M4 (aa 1–450) that lacks the C terminus and the cyclin-binding motif to perform the NF-κB reporter assay as we showed in Figure 1. Our data demonstrated that SAMHD1 WT and M4 similarly suppressed IKKα- or IKKβ-mediated NF-κB activation in HEK293T cells with or without TNF-α treatment (Fig. 8). These results suggest that the C terminus of SAMHD1, including the cyclin-binding motif, is not required for SAMHD1-mediated inhibition of NF-κB activation.

### The KD of IKKα and ULD of IKKβ are required for their interaction with SAMHD1

**Figure 9:**
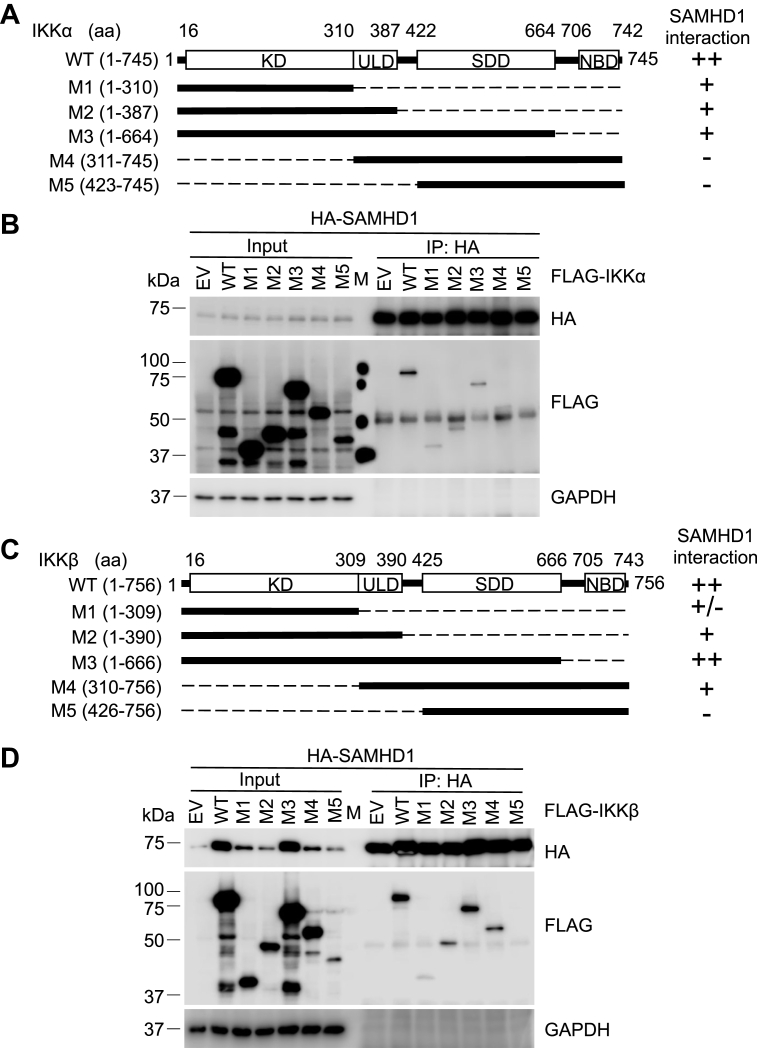
**The KD of IKKα and ULD of IKKβ are required for their interaction with SAMHD1.***A*, schematic diagrams of IKKα WT and truncated mutants (M1-M5). *B*, HEK293T cells were cotransfected with plasmids expressing HA-SAMHD1 or empty vector and FLAG-IKKα WT or FLAG-IKKα truncated mutants (M1 to M5) for 48 h. HA antibody was used for immunoprecipitation (IP). Input and IP samples were analyzed by Western blot. *C*, schematic diagrams of IKKβ WT and truncated mutants (M1-M5). *D*, HEK293T cells were cotransfected with HA-SAMHD1 or empty vector and FLAG-IKKβ WT or FLAG-IKKβ truncated mutants (M1 to M5) for 48 h. HA antibody was used for IP. Input and IP samples were analyzed by Western blot. KD, kinase domain; NBD, NEMO-binding domain; SDD, scaffold dimerization domain; ULD, ubiquitin-like domain.

To map the specific domains of IKKα and IKKβ required for interaction with SAMHD1, a Co-IP assay was performed in HEK293T cells coexpressing WT SAMHD1 and WT IKKα, WT IKKβ, or a series of truncated IKKα or IKKβ mutants (M1-M5) (Fig. 9, *A* and *C*) (21). IKKα M4 and M5 lacking the KD did not interact with SAMHD1, while IKKα WT, M1, M2, M3 containing KD interacted with WT SAMHD1 (Fig. 9, *A* and *B*), suggesting that the KD of IKKα is required for its interaction with SAMHD1. Interestingly, IKKβ M1 interacted only weakly with SAMHD1, while IKKβ WT, M2, M3, and M4 containing the ULD strongly interacted with SAMHD1 (Fig. 9*D*), suggesting that the interaction is mostly dependent on the ULD of IKKβ. Thus, the KD of IKKα and ULD of IKKβ mediate their respective interactions with SAMHD1.

### Exogenous SAMHD1 inhibits the interaction between TAK1 and IKKα or IKKβ

**Figure 10:**
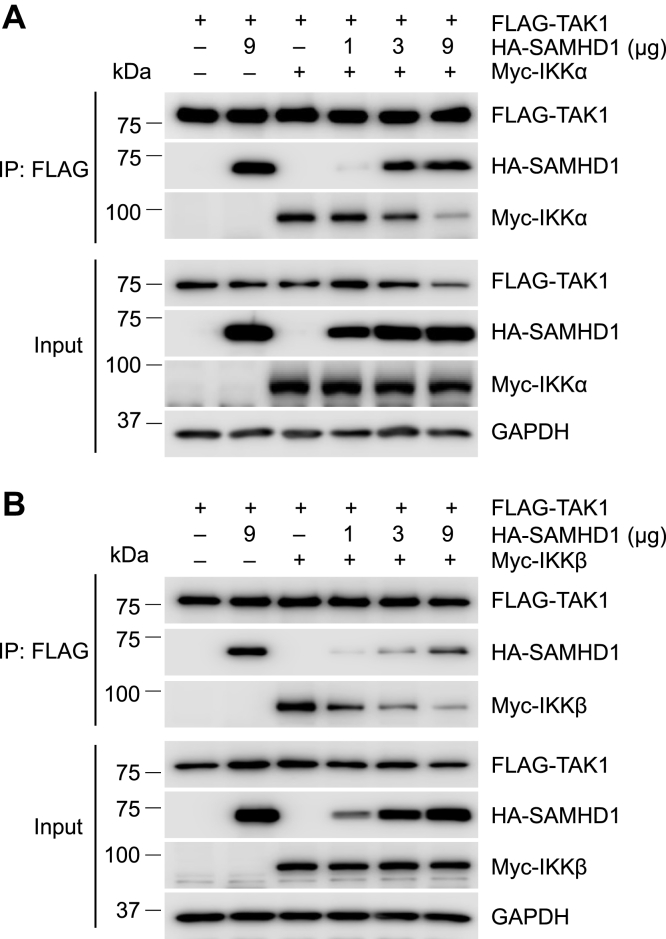
**Exogenous SAMHD1 inhibits the interaction between TAK1 and IKKα or IKKβ.***A* and *B*, HEK293T cells were cotransfected with plasmids expressing FLAG-TAK1 and increasing amounts of HA-SAMHD1 and either Myc-IKKα (*A*) or Myc-IKKβ (*B*). Immunoprecipitation (IP) was performed using FLAG antibody. Input and IP samples were analyzed by Western blot.

To explore the exact mechanism of SAMHD1-mediated inhibition of p-IKKα and p-IKKβ, HEK293T cells were cotransfected with constructs expressing FLAG-TAK1, increasing amounts of HA-SAMHD1 and Myc-IKKα or Myc-IKKβ. Co-IP and immunoblot analysis showed that exogenous SAMHD1 decreases the interaction between TAK1 and IKKα (Fig. 10*A*) or IKKβ (Fig. 10*B*) in a dose-dependent manner. These data suggest that SAMHD1 blocks the TAK1-binding site of IKKα and IKKβ and thereby inhibits phosphorylation of IKKα and IKKβ.

## Discussion

NF-κB activation is critical for efficient innate immune responses to inflammatory stimuli and virus infections (32, 33). We have previously found that SAMHD1 acts as a negative regulatory factor in NF-κB activation and IFN-I induction (14). In this study, we found that SAMHD1 inhibits IKKα- and IKKβ-mediated NF-κB activation. Overexpression of IKKα and IKKβ together induced similar levels of NF-κB activity compared with treatment with TNF-α (Fig. 1*C*), indicating that IKKα and IKKβ could synergistically activate the NF-κB pathway. Moreover, we revealed that SAMHD1 suppressed the phosphorylation of IKK complex proteins, which play an important role in NF-κB activation. Upon inflammatory stimuli treatment or SeV infection, the phosphorylation of IKKα/β reaches a peak and diminishes quickly (Figs. 2 and 3) to potentially avoid sustained and overexuberant inflammatory innate immune responses. Moreover, SAMHD1 KO cells showed an earlier and stronger response to inflammatory stimuli and virus infection compared with THP-1 cells (Figs. 2 and 3), suggesting that SAMHD1 plays an important role in the balance of innate immune responses to inflammatory stimuli and viral infections. The dNTPase activity of SAMHD1 is not required for its suppression of NF-κB activation in dividing HEK293T cells, while it is essential for NF-κB inhibition in nondividing monocytic cells (14, 29), suggesting that the function of SAMHD1 is dependent on cell type or cell differentiation. In this study, we found that SAMHD1 interacts with IKKα and IKKβ and inhibits phosphorylation of IKKα/β/γ in both dividing and nondividing THP-1 cells (Figure 2, Figure 3, Figure 4, Figure 5).

In this study, the SAMHD1 HD domain was found to interact with IKKα and IKKβ (Fig. 7). Our previous results revealed that the SAMHD1 HD domain interacts with IRF7 and their interaction is required for SAMHD1-mediated inhibition of IFN-I activation (14). Together, our data suggest that the HD domain of SAMHD1 plays a vital role in regulating the innate immune response, including the NF-κB and IFN-I pathways. The HD domain of SAMHD1 is responsible for the dNTPase activity, and SAMHD1 containing two amino acid mutations (H206R and D207N, HD/RN) was confirmed to lose its dNTPase activity (2). Our previous results showed that dNTPase activity is necessary for SAMHD1-mediated suppression of NF-κB activation and IFN-I induction in nondividing monocytic cells (29). In this study, we showed that SAMHD1 mutants lacking the HD domain did not interact with IKKα and IKKβ in dividing HEK293T cells (Fig. 7). SAMHD1 nuclear localization is dependent on the nuclear localization signal of SAMHD1 (34, 35) and is not required for SAMHD1-mediated suppression of NF-κB activation and IFN-I induction (29). Because the interaction between SAMHD1 and IKKα or IKKβ is dependent on the HD domain of SAMHD1, and phosphorylation of IKKα/β occurs in the cytoplasm (36), it is unlikely that nuclear localization signal is required for SAMHD1 suppression of IKKα and IKKβ-mediated NF-κB activation. Moreover, our results indicated that the C terminus of SAMHD1 is not required for SAMHD1-mediated inhibition of NF-κB activation (Fig. 8).

**Figure 11:**
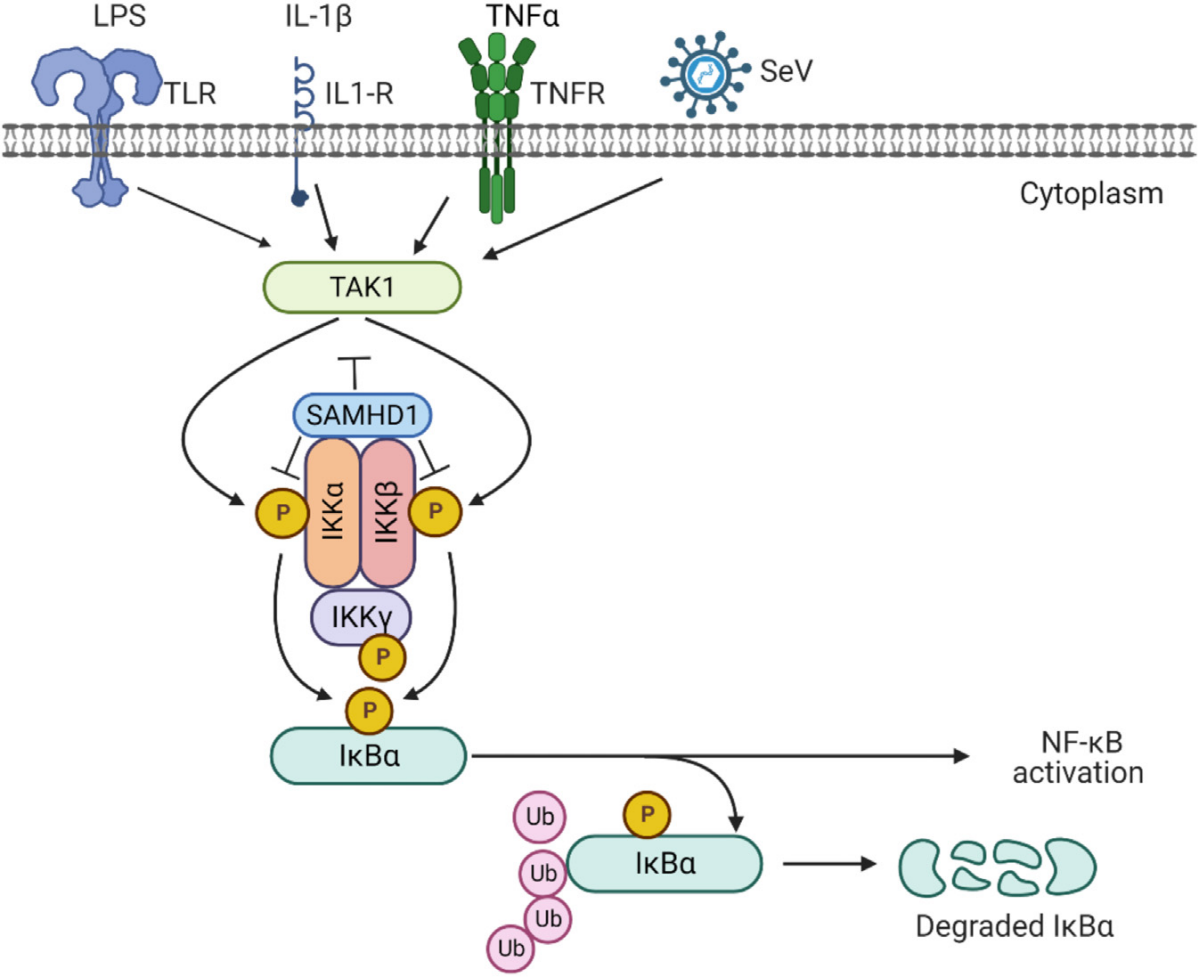
**SAMHD1 inhibits phosphorylation of IKKα/β/γ and interacts with IKKα and IKKβ.** SeV infection or inflammatory stimuli (LPS, TNF-α, or IL-1β) are recognized by corresponding receptors and induce phosphorylation of IKKα/β/γ and IκBα (indicated with a letter P). Cytoplasmic SAMHD1 interacts with the IKKα kinase domain and IKKβ ubiquitin-like domain and disrupts the interaction between TAK1 and IKKα or IKKβ to inhibit phosphorylation of IKKα/β/γ induced by inflammatory stimuli or SeV infection. The NF-κB inhibitory protein IκBα is phosphorylated by IKKα/β and degraded by the proteasome to activate subsequent NF-κB target gene transcription. Thus, SAMHD1 inhibits IKKα- and IKKβ-mediated NF-κB activation. Our findings revealed negative regulatory mechanisms by which SAMHD1 suppresses IκBα phosphorylation. Ub, ubiquitination. This figure was created with BioRender.com.

Furthermore, we found that full-length WT SAMHD1 interacted with IKKα KD and IKKβ ULD, respectively (Fig. 9). Full-length IKKα and IKKβ share 54% amino acid sequence identity, while their KDs show 65% identity (17). Functionally, both IKKα and IKKβ are part of the canonical pathway of NF-κB activation, whereas IKKα is involved in the noncanonical pathway (17). The obvious difference in structure between IKKα and IKKβ is in the orientation of the KD relative to the SDD and ULD as determined by structural studies (37, 38), suggesting pronounced differences between IKKα and IKKβ in their structure and function. The KD and ULD are responsible for the catalytic activity of IKKα and IKKβ, and ULD and SDD are involved in interaction with IκBα (39). Thus, SAMHD1 bound to these domains may result in affecting the kinase activity and the interaction between IκBα and IKKα or IKKβ. Some NF-κB inhibitors such as large tumor suppressor gene 2 (LATS2) and Nemo-like kinase (NLK) disrupt the interaction between TAK1 and IKKβ to inhibit NF-κB activation (40, 41). SAMHD1 binds to IKKα and IKKβ directly, which may prevent the phosphorylation of IKKα and IKKβ by TAK1. On the one hand, our previous study found that SAMHD1 inhibited the phosphorylation of TAK-1 to suppress TRAF6 and TAK1-mediated NF-κB activation (28). On the other hand, SAMHD1 interacts with IKKα and IKKβ directly and disrupts the interaction between TAK1 and IKKα or IKKβ, which may lead to inhibition of the phosphorylation of the IKK complex and IκBα (Fig. 11). Through these two distinct negative regulatory mechanisms, we could further understand the mechanism by which SAMHD1 inhibits the phosphorylation of IκBα.

In our previous published studies on SAMHD1-mediated suppression of NF-κB activation (14, 29), we focused on the interaction between SAMHD1 and the key NF-κB proteins (IκBα, p100/p52, and p102/p50); however, we did not investigate the role of the IKK complex in the pathway. In the current study, we found that SAMHD1 interacts with the IKK complex to inhibit the phosphorylation of IKKα/β/γ and IκBα, thereby revealing negative regulatory mechanisms of SAMHD1 in the inhibition of p-IκBα and NF-κB activation. Thus, our results provide new insights into the inhibitory role of SAMHD1 in NF-κB activation, which is important for antiviral innate response and inflammatory diseases.

## Experimental procedures

### Antibodies

Primary antibodies used in the study and their resources were SAMHD1 (catalog number ab67820, Abcam), Tubulin (ab7291, Abcam), IKKα (2682, Cell Signaling), IKKα (61294S Cell Signaling), IKKβ (8943, Cell Signaling), Phospho-IKKα/β (Ser176/180) (2697, Cell Signaling), IKKγ (2685, Cell Signaling), Phospho-IKKγ (Ser376) (2689, Cell Signaling), IκBα (4814, Cell Signaling), Phospho-IκBα (Ser32/36) (9246, Cell Signaling), GAPDH (AHP1628, Bio-Rad), HA (H6908, Sigma-Aldrich), FLAG (F1804, Sigma-Aldrich), and SeV (PD029, MBL Life Science). Secondary antibodies used were goat anti-mouse IgG (H + L) HRP (W4021) and goat anti-rabbit lgG (H + L) HRP (W401B) (Promega); mouse anti-rabbit IgG (L) (211-032-171) and goat anti-mouse IgG (L) (115-035-174) (Jackson ImmunoResearch Inc).

### Reagents and source

LPS (L6529), puromycin (P8833), and PMA (P8139) were purchased from Sigma-Aldrich. TNF-α (300-01A) and IL-1β (200-01B) were from Peprotech. Cell lysis buffer (9803) was from Cell Signaling. Phosphatase Inhibitor Cocktail 3 (P0044) and Protease inhibitor cocktail (P8340) were from Sigma-Aldrich. Dynabeads Protein G (10004D), Dynabeads Protein A (10001D), SuperSignal West Femto Maximum Sensitivity Substrate (34096), and Pierce BCA Protein Assay (23225) were from Thermo Fisher Scientific. Dual-Luciferase Reporter Assay System (E1910) was from Promega. Nitrocellulose membrane (1620115) was from Bio-Rad. Polyethyleneimine (PEI) (24313-2) was purchased from Polysciences. Purified recombinant IKKα (TP761707) and IKKβ (TP750220) were purchased from Origene.

### Cell culture

THP-1 control, THP-1 SAMHD1 KO, THP-1 Lvx, and THP-1 SAMHD1 KI cell lines were described previously (30). The cells were cultured in RPMI 1640 (ATCC) with 10% fetal bovine serum, 100 U/ml penicillin, 100 μg/ml streptomycin, and 1 μg/ml puromycin. HEK293T cells were grown in Dulbecco’s modified Eagle’s medium with 10% fetal bovine serum, 100 U/ml penicillin, and 100 μg/ml streptomycin as described (29). All cell lines were at 37 °C, 5% CO_2_ and confirmed free from mycoplasma contamination using the universal mycoplasma detection kit (ATCC 30-101-2K). Trypsin-EDTA (0.25%) (25200072), Dulbecco’s modified Eagle’s medium (11965092), Opti-MEM (31985070), and Penicillin-Streptomycin (15140122) were bought from Gibco. Fetal Bovine Serum (S1150H) was bought from R&D Systems.

### Plasmids

Plasmids (pRK based) encoding HA-tagged SAMHD1 WT and truncated mutants (M1, M2, M3, M4, M6, and M7) (see schematic diagrams in Fig. 7*A*) were reported or constructed as described (14). The coding regions of human IKKα (transcript ID: NM_001278.5), IKKβ (transcript ID: NM_001556.3), and IKKγ gene (transcript ID: NM_001321396.3) were amplified by PCR using plasmids pCR-Myc-hIKKα, pcDNA-FLAG-IKKβ, and pcDNA-FLAG-IKKγ, respectively, and inserted into the HindIII and KpnI sites of pN3-3xFLAG-Control (Addgene, plasmid #107717). pCR-Myc-hIKKα was constructed by PCR amplification of human IKKα cDNA and replacing mouse IKKα cDNA in pCR-Myc-IKKα (Addgene, plasmid #19739). pcDNA-FLAG-IKKβ and pcDNA-FLAG-IKKγ were kind gifts from Dr Shuliang Chen (Wuhan University, China). pN3-3xFLAG-IKKα M1 (aa 1–310), M2 (aa 1–387), M3 (aa 1–664), M4 (aa 311–745), and M5 (aa 423–745), as well as pN3-3xFLAG-IKKβ M1(aa 1–309), M2 (aa 1–390), M3 (aa 1–666), M4 (aa 310–756), and M5 (aa 426–756) were constructed using full-length IKKα or IKKβ as a template for PCR amplification. The plasmid encoding FLAG-TAK1 was constructed based on pRK-HA-TAK1 (28). Sequence of all constructs was confirmed by Sanger sequencing.

### SeV infection, treatment with inflammatory stimuli and PMA

SeV was propagated in specific pathogen-free 10-day embryonated chicken eggs (Charles River Laboratories) and titered on LLCMK2 cells. SeV infection of THP-1 control, THP-1 SAMHD1 KO, THP-1 Lvx, and THP-1 SAMHD1 KI cell lines was conducted as described (14). THP-1 control and SAMHD1 KO cells were treated with recombinant IL1-β (10 ng/ml), TNF-α (10 ng/ml), or LPS (100 ng/ml) for the indicated times as reported (14, 29). THP-1 control cells, THP-1 SAMHD1 KO cells, THP-1 Lvx cells, and THP-1 SAMHD1 KI cells were treated with PMA (30 ng/ml) as described (29).

### Dual luciferase assays

HEK293T cells were transfected with pNF-κB-luciferase, pRL-TK-renilla, and indicated plasmids for 48 h using PEI. For dose response experiments, an empty vector was used to maintain the same amount of the total plasmid in each transfection. The cells were lysed by passive lysis buffer, and luciferase was measured by VICTOR Nivo Multimode Microplate Reader using the method described (28).

### Western blot

The cells were lysed in cell lysis buffer as described (28). Protein was electrophoretically separated by SDS-PAGE and transferred onto nitrocellulose membranes. Membranes were blocked in 5% nonfat dry milk and then incubated with primary antibody, followed by HRP-labeled secondary antibody. The membrane was developed with ECL and visualized using Odyssey Fc Imager. GAPDH or tubulin was used as a control to normalize loading for quantification by densitometry.

### Co-IP assay

The THP-1 control cells or PMA-differentiated THP-1 control cells were harvested and lysed in the cell lysis buffer. SAMHD1 antibody (4 μg) and Dynabeads protein G were used for IP. The same amounts of mouse IgG were used as the negative control. The bound beads were washed with PBS and 0.1% Tween three times and then boiled in protein loading buffer (31). HEK293T cells were collected after 48 h transfection and lysed in the cell lysis buffer for 10 min. HA antibody or FLAG antibody and Dynabeads protein G were used for IP and corresponding mouse IgG or rabbit IgG was used as the negative control. The bound beads were washed with washing buffer (50 mM Tris-HCl [pH 7.4], 150 mM NaCl, 1% NP-40, and 0.25% sodium deoxycholate). Input and IP samples were analyzed by Western blot as described (29).

### Recombinant proteins and *in vitro* pull down

Recombinant SAMHD1 was purified as described (42) and the pull-down assay was performed as described (14). SAMHD1 and IKKα or IKKβ were precleared with Dynabeads Protein A for 30 min and then incubated with Dynabeads Protein A and SAMHD1 antibody, IKKα antibody (61294S, Cell Signaling) or IKKβ antibody in cell lysis buffer with 0.375% CHAPS overnight at 4 °C. The beads were washed with a buffer containing 50 mM Tris-HCl PH 8.0, 150 mM NaCl, 1% Triton X-100, and 0.5% sodium deoxycholate. The input and IP samples were analyzed by Western blot.

### Statistical analysis

The results were presented as mean ± SD. Data were analyzed using the GraphPad Prism software and nonparametric *t* tests were performed for statistical comparison between groups. Statistical differences were considered significant at a value of *p* < 0.05.

## Data availability

All data are contained within the article.

## Supporting information

This article contains supporting information.

## Conflict of interest

The authors declare that they have no conflicts of interest with the contents of this article.
