# Supplementary material for: The host antiviral protein SAMHD1 suppresses NF-κB activation by interacting with the IKK complex during inflammatory responses and viral infection
Source: J Biol Chem. 2023 Apr 24;299(6):104750. doi: 10.1016/j.jbc.2023.104750 (PMC10318468; doi:10.1016/j.jbc.2023.104750)
Supplement: Supporting Figures S1–S3 [file mmc1.pdf]

Yang *et al*. The host anti-viral protein SAMHD1 suppresses NF-κB activation by interacting with the IKK complex during inflammatory responses and viral infection.

Supporting information

Figure S1. SAMHD1 inhibits phosphorylation of IKKα/β/γ induced by TNF-α or IL-1β. (A and B) THP-1 control cells and THP-1 SAMHD1 KO cells were treated with TNF-α (10 ng/mL) (A) or IL-1β (10 ng/mL) (B) for the indicated times or mock-treated. The expression levels of SAMHD1, IKKα/β/γ, p- IKKα/β, p-IKKγ, IκBα, p- IκBα, and tubulin were measured by Western blot. Tubulin was used as a loading control.

Figure S2. PMA induces phosphorylation of IKKα/β. THP-1 control cells and THP-1 SAMHD1 KO cells were treated with PMA (30 ng/mL) for 1-48 h or mock-treated. The expression levels of indicated protein were measured by Western blot. Tubulin was used as a loading control.

Figure S3. Endogenous SAMHD1 interacts with IKKα and IKKβ. (A and B) THP-1 control cells (A) and PMA-differentiated THP-1 cells (B) were treated with LPS (100 ng/mL) for the indicated times or mock-treated. Immunoprecipitation was performed with SAMHD1 antibody, Mouse IgG was used as a negative control. The indicated proteins were detected by Western blot. The relative IKKα and IKKβ levels were quantified by densitometry analysis and the IgG control was set as 1.

Fig. S1

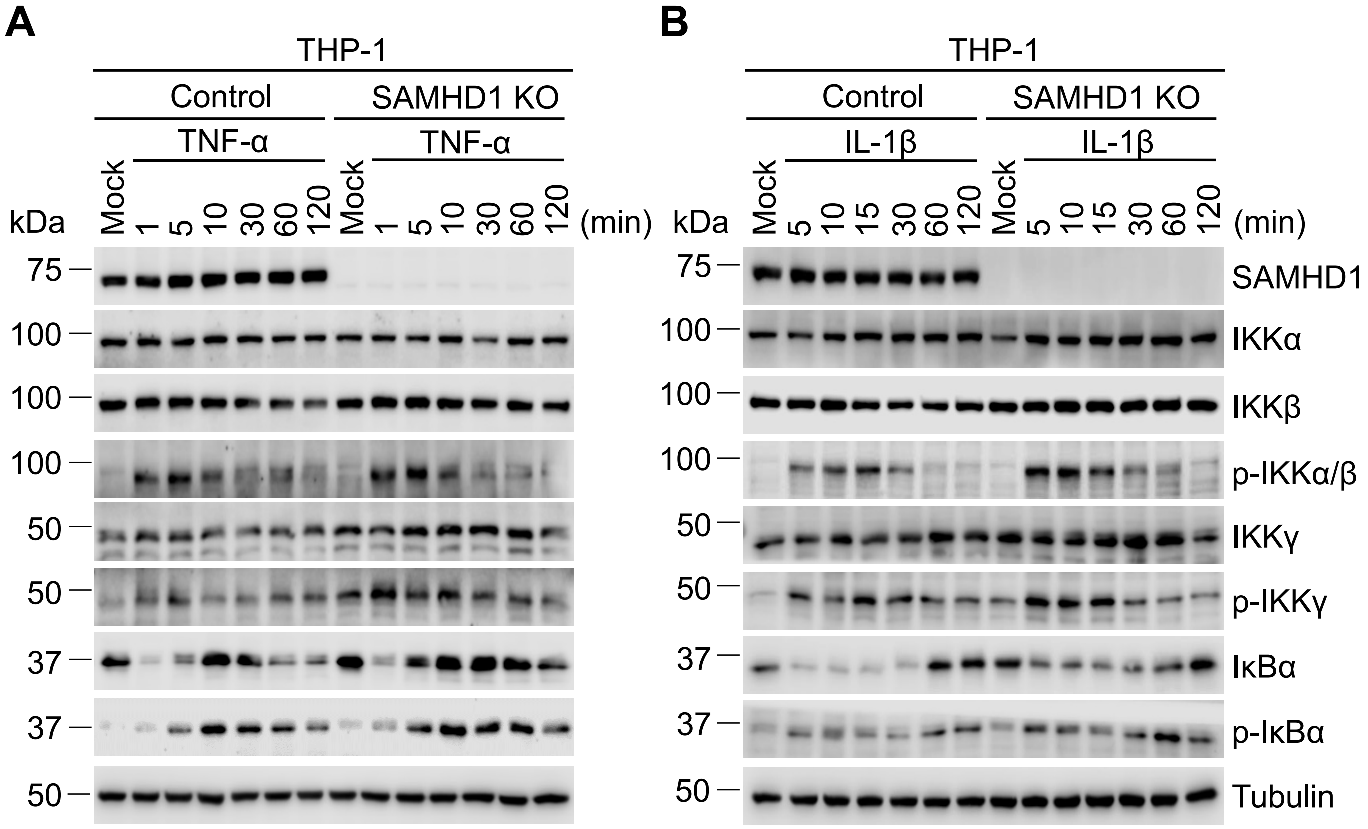
A
THP-1
Control
SAMHD1 KO
TNF-α
TNF-α
Mock 1 5 10 30 60 120 Mock 1 5 10 30 60 120 (min)
kDa
75
100
100
100
50
50
37
37
50
B
THP-1
Control
SAMHD1 KO
IL-1β
IL-1β
Mock 5 10 15 30 60 120 Mock 5 10 15 30 60 120 (min)
kDa
75
100
100
100
50
50
37
37
50
SAMHD1
IKKα
IKKβ
p-IKKα/β
IKKγ
p-IKKγ
IκBα
p-IκBα
Tubulin

Fig. S2

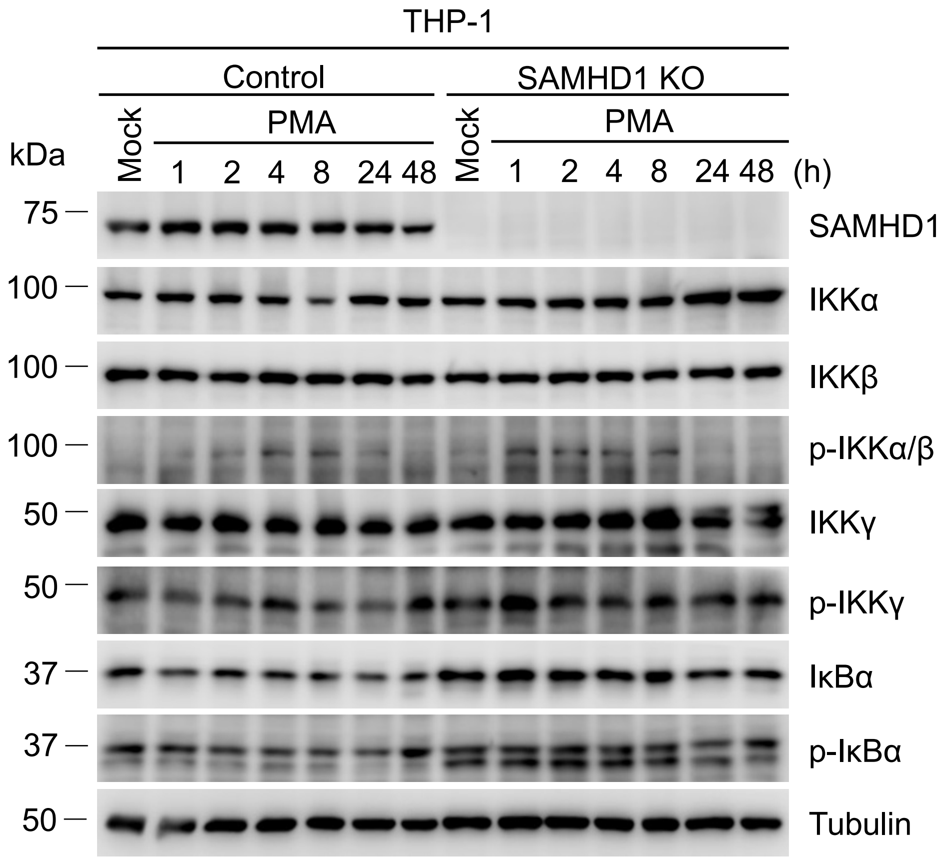
THP-1
Control
SAMHD1 KO
Mock
PMA
Mock
PMA
kDa
1
2
4
8
24
48
1
2
4
8
24
48
(h)
75
100
100
100
50
50
37
37
50
SAMHD1
IKKα
IKKβ
p-IKKα/β
IKKγ
p-IKKγ
IκBα
p-IκBα
Tubulin

Fig. S3

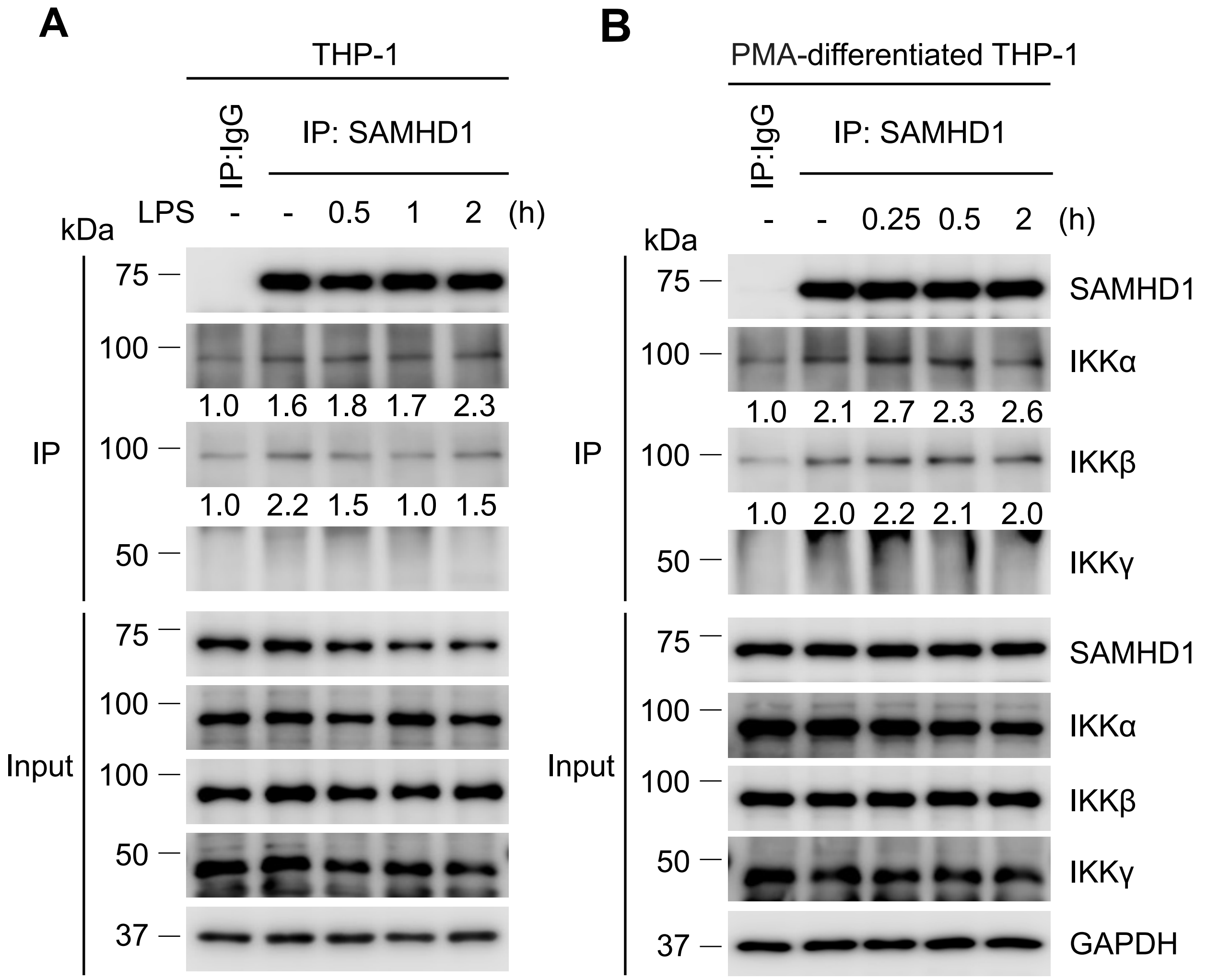
A
THP-1
IP:IgG
IP: SAMHD1
LPS - - 0.5 1 2 (h)
kDa
75
100
1.0 1.6 1.8 1.7 2.3
100
1.0 2.2 1.5 1.0 1.5
50
IP
75
100
100
50
37
Input
B
PMA-differentiated THP-1
IP:IgG
IP: SAMHD1
- - 0.25 0.5 2 (h)
kDa
75
SAMHD1
100
IKKα
1.0 2.1 2.7 2.3 2.6
100
IKKβ
1.0 2.0 2.2 2.1 2.0
50
IKKγ
IP
75
SAMHD1
100
IKKα
100
IKKβ
50
IKKγ
37
GAPDH
Input
